# Post-COVID Neurodegeneration: A Puerto Rican Case of Rapidly Fatal Creutzfeldt-Jakob Disease

**DOI:** 10.7759/cureus.93565

**Published:** 2025-09-30

**Authors:** Francisco J Lopez-Font, Carlos A Vega-Mena, Antonella Jimenez, Ramón Scharbaai-Vázquez, Ernesto García-Santiago

**Affiliations:** 1 Medicine, San Juan Bautista School of Medicine, Puerto Rico, USA; 2 Microbiology, San Juan Bautista School of Medicine, Puerto Rico, USA; 3 Family Medicine, Hospital Menonita, Caguas, USA

**Keywords:** cerebral mri, covid-19, creutzfeldt jakob disease, csf protein 14-3-3, post-covid-19 sequelae

## Abstract

COVID-19 is a disease caused by the SARS-CoV-2 virus, and infection typically leads to the development of respiratory symptoms and illness. Neurodegenerative disorders following infection with SARS-CoV-2 have been reported. We report the case of a previously healthy 58-year-old native Puerto Rican man with a history of COVID-19 vaccination who developed Creutzfeldt-Jakob Disease (CJD) 14 days after a COVID-19 illness. The clinical features and disease course aligned with those described in post-COVID-19 CJD cases documented in the literature. Magnetic resonance imaging (MRI) revealed prominent T2/fluid attenuated inversion recovery (FLAIR) signal abnormalities within the entire cortex of both cerebral hemispheres. Cerebrospinal fluid (CSF) analysis revealed elevated 14-3-3 gamma concentrations, along with a positive real-time quaking-induced conversion (RT-QuIC), confirming the diagnosisofCJD. While further investigation is required to elucidate the relationship between these two pathologies, our study underscores the importance of close monitoring post COVID-19, as cognitive sequelae can be indicators of rapid neurodegenerative entities such as sporadic CJD (sCJD).

## Introduction

Creutzfeldt-Jakob Disease (CJD) is a rare, rapidly progressive, and fatal prion disorder characterized by misfolding of the prion protein, leading to widespread neurodegeneration [1]. The disease typically presents with rapidly progressive dementia, visual disturbances, ataxia, and myoclonus, with a mean survival of four to six months [1-3]. Recent studies have raised concerns about a potential association between COVID-19 infection and the pathogenesis of neurodegenerative diseases, including Alzheimer’s and Parkinson’s disease [4-7]. Cases of CJD emerging after COVID-19 infection contribute to evidence that COVID-19 could influence the progression of neurodegenerative disease. 

We report the case of a previously healthy, vaccinated 58-year-old native Puerto Rican male patient who developed neurodegenerative signs two weeks after an infection with COVID-19.

## Case presentation

A previously healthy 58-year-old native Puerto Rican male patient had a positive SARS-CoV-2 antigen test in August of 2022 after experiencing moderate flu-like symptoms, including chest tightness, dry cough, fever, malaise, and generalized weakness. Two weeks afterwards, the patient began to experience difficulty completing basic tasks, suffering from “brain fog,” in addition to fatigue, frequent nightmares, and episodes of vertigo. Over the next two weeks, the patient’s symptoms worsened, resulting in recurrent episodes of vertigo, visual disturbances, tremors, cerebellar ataxia, and difficulty concentrating. Consequently, the patient presented to the emergency room (ER) in September 2022.

Initial evaluation in the ER revealed an alert, oriented, and communicative patient with a chief complaint of worsening neuropsychiatric symptoms. Past medical history was unremarkable, besides a recent COVID-19 illness four weeks prior. The patient had previously received two doses of the Moderna COVID-19 vaccine without complications. The wife, who accompanied the patient, also claimed to have noticed some mood and behavior alterations over the past few weeks. Physical examination of the patient revealed gait abnormalities and abnormal finger-to-nose testing bilaterally. It was determined to hospitalize the patient in order to perform additional diagnostic evaluations.

**Figure 1:**
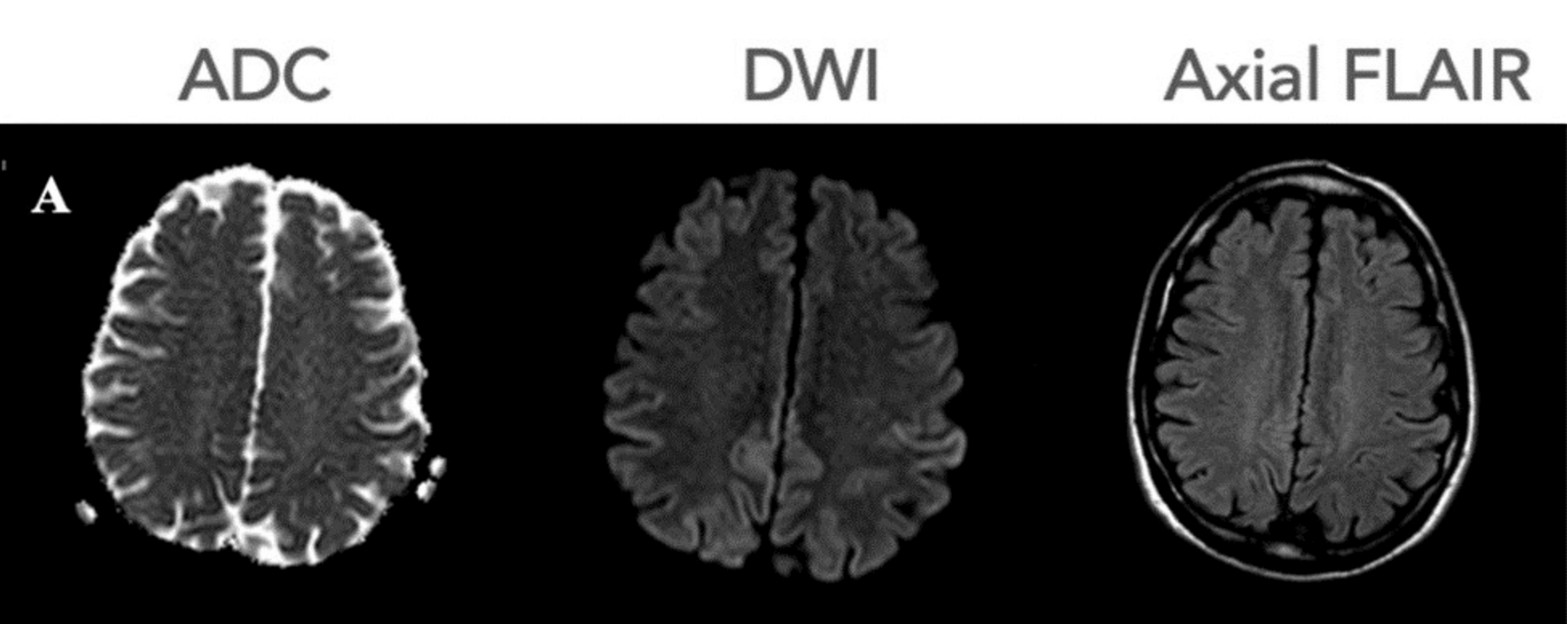
Brain magnetic resonance imaging (MRI) at the patient’s first visit (September 22, 2022) showing subtle abnormal signal intensity throughout most of the parietal cortex bilaterally. Some of these areas were associated with subtle restricted diffusion. ADC: apparent diffusion coefficient map; DWI: diffusion-weighted image; FLAIR: fluid attenuated inversion recovery

Magnetic resonance imaging (MRI) scan of the brain showed subtle abnormal signal intensity throughout most of the parietal cortex bilaterally, with nonspecific periventricular white matter T2 hyperintensities that favored sequelae of chronic small vessel disease but showed no masses, hemorrhage, or acute/subacute ischemia (Figure 1).

Additionally, a magnetic resonance venography (MRV) scan showed normal patent dural venous vessels. Lumbar puncture was performed to rule out infectious etiologies. Initial cerebrospinal fluid (CSF) analysis was normal, and while waiting for additional CSF results, empiric therapy for infectious and/or autoimmune encephalitis with intravenous immunoglobulin and IV methylprednisolone was started. After obtaining negative results for many possible viral encephalitis etiologies, a sample of the patient’s CSF was also sent to the National Prion Disease Pathology Surveillance Center (NPDPSC) for the possibility of CJD.

**Figure 2:**
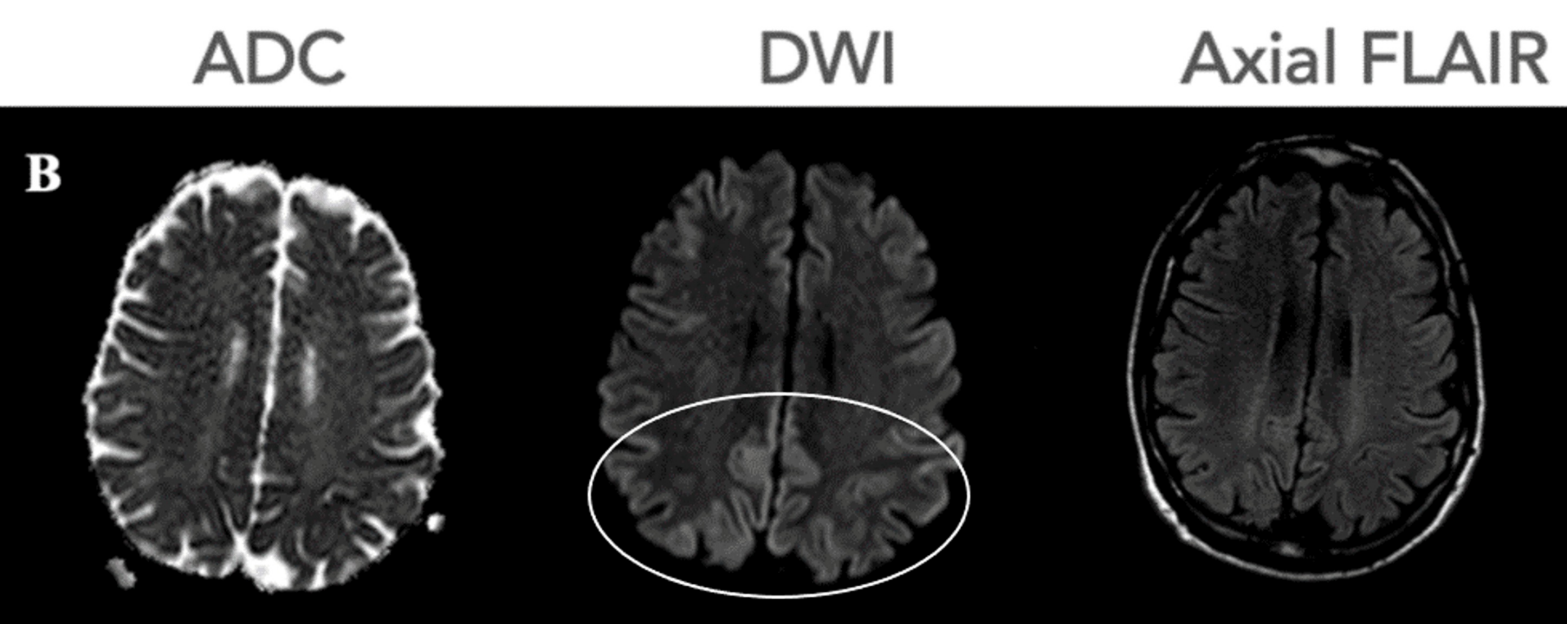
A second brain magnetic resonance imaging (MRI) performed seven days after the initial scan showed more extensive cortical hyperintensities on DWI (circle). ADC: apparent diffusion coefficient map; DWI: diffusion-weighted image; FLAIR: fluid attenuated inversion recovery

Over the subsequent weeks, the patient deteriorated rapidly, developing occasional episodes of myoclonic jerks, seizures, weakness, speech apraxia, and dysarthria. A repeat brain MRI (Figure 2) several days after admission revealed more extensive cortical-based abnormalities involving the bilateral frontal, parietal, and posterior temporal lobes. Furthermore, in response to the onset of seizures, an electroencephalography (EEG) was conducted. The initial EEG revealed diffuse intermixed theta and delta activity throughout the trace with diffuse slowing, suggestive of severe encephalopathy likely due to an infectious, post-ictal, vascular, or metabolic etiology.

**Figure 3:**
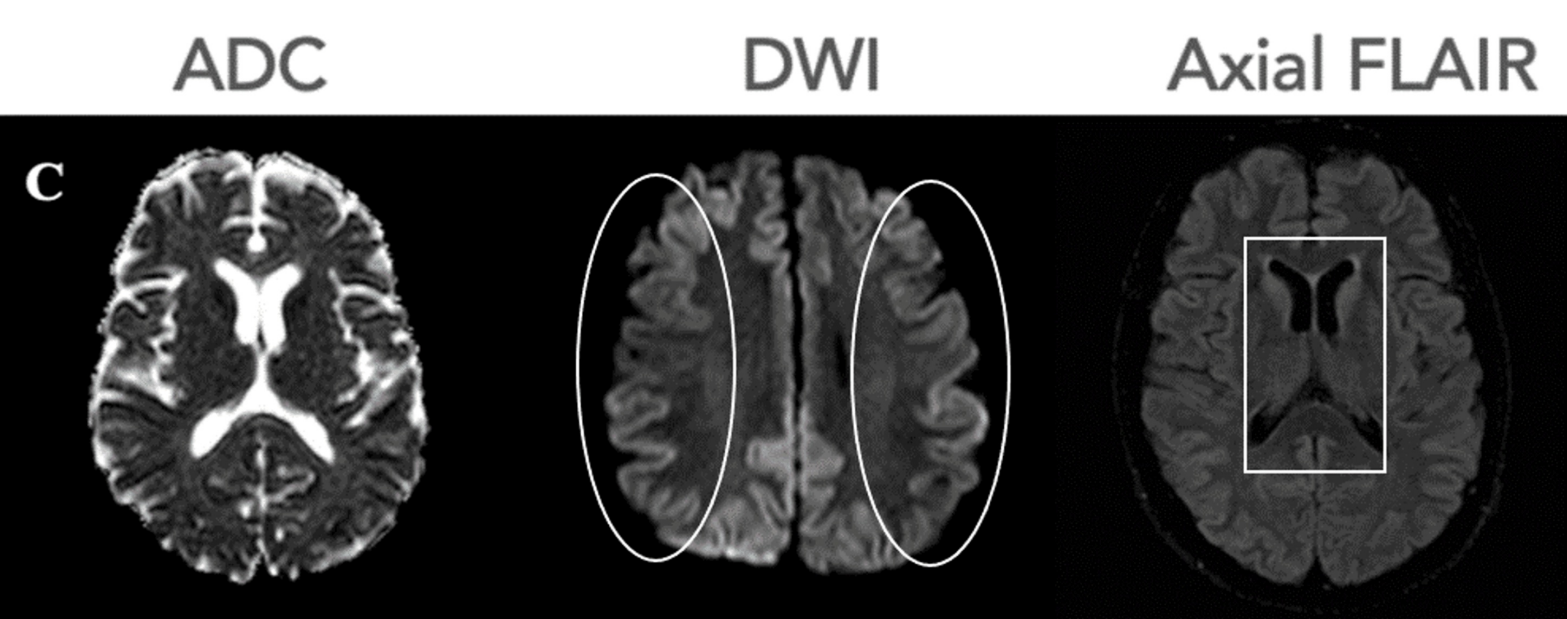
A third brain MRI was obtained on October 11, 2022. The entire cortex of both cerebral hemispheres showed intermediate to high T2/FLAIR signal intensity in the frontal, parietal, and temporal lobes, with similar changes now evident in the caudate, putamina, and thalami as noted on the axial FLAIR (square). Signal hyperintensity of the cortical gyri (cortical ribbon sign), typical of Creutzfeldt-Jakob disease, is now more evident on DWI (circles). ADC: apparent diffusion coefficient map; DWI: diffusion-weighted image; FLAIR: fluid attenuated inversion recovery

By mid-to-late October, the patient was suffering from severe confusion and disorientation, generalized myoclonus, akinetic mutism, and global neurocognitive decline. The patient was treated with levetiracetam. A third brain MRI scan (Figure 3) showed cortical-based high/intermediate T2/fluid attenuated inversion recovery (FLAIR) signal intensity with restricted diffusion involving the bilateral frontal, parietal, and temporal lobes, as well as the insula. Additionally, intermediate T2/FLAIR signal intensity involving both amygdalae and hippocampi and high T2/FLAIR bilateral hyperintensity involving lentiform nuclei, caudate nuclei, and thalami were noted. A repeat EEG demonstrated diffuse slowing with intermixed periodic lateralized epileptiform discharges (PLEDs), which are nonspecific and not considered characteristic of CJD. The prior MRI, however, demonstrated cortical ribboning, a finding more typical of CJD. 

Finally, the results of the CSF biomarker analysis for prion disease, obtained from the NPDPSC in late October, indicated that the patient had a > 98% likelihood of prion disease based on a positive real-time quaking-induced conversion (RT-QuIC), along with elevated T-tau protein and 14-3-3 gamma protein concentrations (Table 1).

**Table 1:** CSF prion panel RT-QuIC: real-time quaking-induced conversion

Parameter	Result	Reference Range
RT-QuIC	Positive	Negative
T-tau protein	17,293 pg/mL	0 – 1,149 pg/mL
14-3-3 gamma	60, 801 AU/mL	< 30 – 1,999 AU/mL

Given the patient’s clinical and laboratory findings, the patient met all of the required criteria for a “probable diagnosis” of sporadic CJD (sCJD) according to the Centers for Disease Control and Prevention’s (CDC) diagnostic guidelines [8]. The patient was ultimately transferred to hospice and died in early 2023, approximately five months after symptom onset.

## Discussion

To our knowledge, this case represents the most rapid onset of neuropsychiatric symptoms and memory impairment associated with CJD following COVID‑19 infection, with symptoms beginning 14 days post infection. Additionally, to our knowledge, this is the first reported case of post-COVID CJD in a native Puerto Rican patient. The early neurological symptoms observed in our case are consistent with those reported in other cases of post-COVID CJD, including cognitive decline [9], confusion [10,11], gait abnormalities [11,12], myoclonus [12], ataxia [13,14], apraxia [15], vertigo, fatigue, and disorientation [16,17]. 

Since COVID-19 has been associated with a wide range of central nervous system (CNS) complications [6-8], distinguishing between CNS sequelae of COVID-19 and post-COVID CJD is crucial for timely intervention, as they differ in pathophysiology, clinical presentation, and prognosis. Neurological complications typically occur during or immediately after acute COVID-19 infection or as part of a post-acute sequelae (also known as "long COVID") and can improve or stabilize over time [6]. Post-COVID CJD, on the other hand, typically emerges months after the acute COVID-19 infection and is characterized by a rapid and often fatal progression. A hallmark feature in all post-COVID CJD cases reviewed is the abrupt onset of cognitive decline [9-17].

Although there is no direct evidence that COVID-19 causes or triggers CJD, the documented case reports of individuals developing or experiencing rapid progression of CJD shortly after COVID-19 infection suggest a potential association between the two conditions. In the cases reviewed, all patients experienced symptom durations ranging from a few weeks to several months. In contrast, typical sCJD often progresses over four to six months, though some subtypes may progress more quickly or slowly [1-3]. Potential triggers should be considered separately. In this case, the temporal proximity of CJD onset to SARS-CoV-2 infection raises the possibility that virus-induced immune dysregulation may have accelerated underlying neurodegenerative processes. However, current evidence supports only a temporal association, not causality, and population-based studies have shown no overall increase in CJD incidence during the pandemic [18]. Ongoing surveillance and prion-focused research remain essential.

Given that the patient also received two doses of the Moderna COVID-19 vaccine, vaccination must also be considered as a potential factor. While isolated neurological complications have been reported shortly after Moderna vaccination [19], no epidemiological evidence to date supports a link between COVID-19 vaccines and CJD or other prion diseases [18,20].

In summary, the patient’s rapidly progressive dementia with cerebellar signs, MRI evidence of cortical ribboning and basal ganglia hyperintensity, and CSF findings of a positive RT-QuIC assay with elevated total tau and 14-3-3 gamma proteins meet the CDC criteria for probable sCJD.

## Conclusions

This case highlights the unusually rapid progression of CJD symptoms following COVID-19 infection. While the link between SARS-CoV-2 and CJD remains unclear, clinicians should remain vigilant. New or rapidly worsening cognitive decline after COVID-19 may signal the onset of a rapidly progressive neurodegenerative disorder like CJD, warranting prompt evaluation and monitoring.
